# Apparent Diffusion Coefficient as an Early Predictive Factor of Local and Overall Response to Treatment with Androgen Deprivation Therapy and Radiotherapy in Patients with Prostate Cancer

**DOI:** 10.3390/cancers17050762

**Published:** 2025-02-24

**Authors:** Victor Duque-Santana, Julio Fernandez, Ana Diaz-Gavela, Manuel Recio, Luis L. Guerrero, Marina Peña, Sofia Sanchez, Fernando López-Campos, Israel J. Thuissard, Cristina Andreu-Vázquez, David Sanz-Rosa, Vérane Achard, Alfonso Gómez-Iturriaga, Víctor Díez, Barbara A. Jereczek-Fossa, Elia Del Cerro, Felipe Couñago

**Affiliations:** 1 Department of Radiation Oncology, Hospital Universitario Quirónsalud Madrid y Hospital La Luz, 28223 Madrid, Spain; anadiazgavela@gmail.com (A.D.-G.); luisleoguerrero01@gmail.com (L.L.G.); marina.pena@quironsalud.es (M.P.); sofia.sanchez@quironsalud.es (S.S.); elia.delcerro@quironsalud.es (E.D.C.); 2 Department of Medicine, Faculty of Medicine, Health and Sports, European University of Madrid, 28108 Madrid, Spain; manuel.recio@quironsalud.es (M.R.); israeljohn.thuissard@universidadeuropea.es (I.J.T.); david.sanz@universidadeuropea.es (D.S.-R.); felipe.counago@genesiscare.es (F.C.); 3 Department of Radiology, Hospital Universitario Quirónsalud Madrid, 28223 Madrid, Spain; julio.fernandezma@quironsalud.es; 4 Department of Radiation Oncology, Hospital Universitario Ramon y Cajal, 28034 Madrid, Spain; fernando_lopez_campos@hotmail.com; 5 Department of Veterinary Medicine, Faculty of Biomedical and Health Sciences, European University of Madrid, 28108 Madrid, Spain; cristina.andreu@universidadeuropea.es; 6 Department of Radiation Oncology, Institut Bergonié, 33000 Bordeaux, France; v.achard@bordeaux.unicancer.fr; 7 Department of Radiation Oncology, Hospital Universitario de Cruces, 48903 Barakaldo, Spain; agomeziturriaga@gmail.com; 8 Department of Urology, Hospital Universitario Quirónsalud Madrid, 28223 Madrid, Spain; vdiez@quironsalud.es; 9 Department of Oncology and Hemato-Oncology, University of Milan, 20122 Milan, Italy; barbara.jereczek@ieo.it; 10 Department of Radiation Oncology, IEO European Institute of Oncology IRCCS, 20141 Milan, Italy; 11 Department of Radiation Oncology, Hospital San Francisco de Asís y La Milagrosa, GenesisCare, 28002 Madrid, Spain

**Keywords:** multiparametric magnetic resonance imaging, apparent diffusion coefficient, prostate cancer, radiotherapy, androgen deprivation therapy

## Abstract

**Background/Objectives**: To analyze the predictive value of the apparent diffusion coefficient (ADC) in patients with prostate cancer (PCa) treated with radiotherapy (RT) and androgen deprivation therapy (ADT). **Methods**: Retrospective study of patients with high-risk, very high-risk, or unfavorable intermediate-risk PCa who received RT and ADT between 2008 and 2019 and underwent multiparametric magnetic resonance imaging mpMRI) at 6 months post-RT. Differences in ADC values were compared between patients with and without progression and/or local recurrence. Receiver operating characteristic (ROC) curves were used to obtain ADC cutoffs for predicting 10-year progression-free-survival (PFS) and local recurrence-free survival (LRFS). **Results**: We evaluated 98 patients (73 [74.5%] high-risk). Over a mean ± SD follow-up of 95.36 ± 30.54 months, 19 patients (19.4%) progressed; at 10 years, PFS was 75.6%, LRFS 93.8%, metastasis-free survival 85.5%, and overall survival 89.5%. Post-RT ADC was significantly lower in patients with local recurrence (1.09 ± 0.18 vs. 1.30 ± 0.20 × 10^−3^ mm^2^/s, *p* = 0.020) and progression (1.23 ± 0.20 vs. 1.30 ± 0.21 × 10^−3^ mm^2^/s, *p* = 0.004). ROC analysis identified a post-RT ADC cutoff of 1.11 × 10^−3^ mm^2^/s for local recurrence (area under curve [AUC] 0.843, sensitivity 89.4%, positive predictive value [PPV] 98.8%). The cutoff for progression was 1.24 × 10^−3^ mm^2^/s (AUC0.705, sensitivity 72.2%, PPV87.7%). Patients with a post-RT ADC value below and above 1.11 × 10^−3^ mm^2^/shad a 10-year LRFS of 66.8% and 97.7%, respectively (HR: 25.04 [2.58–242.92], *p* < 0.001). The corresponding rates for 10-year PFS were 58.6% and 85.6% in patients with post-RT ADC values below and above 1.24 × 10^−3^ mm^2^/s (HR: 2.916 [1.113–7.644], *p* = 0.015). In the multivariate analysis, a post-treatment ADC value ≤ 1.24 × 10^−3^ mm^2^/s was a significant prognostic factor for a lower PFS (HR: 3823 [1371–10,657], *p* = 0.010). **Conclusions**: This is the first study to show that post-RT ADC can be a predictive factor of local recurrence in PCa treated with RT and ADT. Moreover, this long-term study demonstrates its value as a predictive factor of progression in PCa treated with RT and ADT.

## Simple Summary

The article entitled Apparent diffusion coefficient as an early predictive factor of local and overall response to treatment with androgen deprivation therapy and radiotherapy in patients with prostate cancer analyzes the predictive value of the apparent diffusion coefficient in patients with prostate cancer treated with radiotherapy and androgen deprivation therapy. This article shows how the ADC value obtained from multiparametric magnetic resonance imaging after radiotherapy can be used as a predictor of local recurrence and progression and may help to select patients at higher risk of local relapse or any progression who could benefit from a closer follow-up and treatment escalation.

## 1. Introduction

Prostate cancer is the second most common cancer in the world [1]. Treatment options include surgery and radiotherapy (RT) alone or in combination with androgen deprivation therapy (ADT). Similar long-term outcomes have been described for these options [2].

Multiparametric magnetic resonance imaging (mpMRI) is increasingly being used to diagnose and evaluate prostate cancer [3]. Compared with digital rectal exam (DRE), it provides a more accurate picture of local disease extension, has stronger correlations with the Gleason score, and is associated with lower interobserver variability [4]. Its use as a diagnostic tool has led to changes in staging and risk classification. In patients with prostate cancer diagnosed by DRE, for example, mpMRI led to a change in TNM stage in 89.39% of cases and in risk classification in 25.7% [5].

One of the main parameters used in mpMRI is the apparent diffusion coefficient (ADC), a quantitative metric derived from diffusion-weighted (DW) MRI images sequences. The ADC reflects the in vivo motion of water molecules, which may be restricted in certain pathological conditions such as tumors and other lesions. The use of ADC values in prostate cancer has been associated with improved tumor detection, characterization, and staging [6,7,8].

It remains to be determined whether combination therapy with RT and ADT affects mpMRI ADC values in patients with prostate cancer and whether these values might be early prognostic markers of treatment response in this setting. Just one study, published in 2014 and with a very short follow-up (40 months), suggested that ADC values might be useful for the early prediction of treatment response after RT [9].

The aim of this study was to examine the value of post-treatment ADC as an early predictive factor of treatment response in patients with prostate cancer treated with RT and ADT.

The conclusions of the manuscript will note that post-RT ADC values are predictive of local recurrence in patients with intermediate- and high-risk prostate cancer treated with RT and ADT. Moreover, this long-term study shows that post-RT ADC values could be used as an early predictive factor of treatment response in patients with prostate cancer treated with RT and ADT.

## 2. Materials and Methods

### 2.1. Patient Selection

The protocol for this study was approved by the ethics committee at Quironsalud University Hospital, on 14 November 2023 (EO277-23_HUQM). This was a retrospective study of patients diagnosed with unfavorable intermediate-risk, high- or very high-risk prostate cancer treated with RT and ADT between 2008 and 2019.

The inclusion criteria were male patients aged >18 years; a diagnosis of intermediate-, high-, or very high-risk prostate adenocarcinoma according to the National Comprehensive Cancer Network (NCCN) guidelines; treatment with RT and ADT; availability of 6 months post-RT mpMRI scans; and sufficient data with which to calculate tumor ADC values.

Exclusion criteria were initial treatment with radical prostatectomy; a diagnosis of low-risk or favorable intermediate-risk prostate adenocarcinoma; not receiving combined RT and ADT; not undergoing mpMRI after completion of RT, and the inability to calculate tumor ADC values.

Based on previous reports describing the relationship between ADC values post-treatment and clinical outcomes, we estimated a minimum requirement of 36 patients (6 with recurrence and 30 without) to detect differences in ADC values after RT, achieving a statistical power of 80% and a confidence level of 95%. Following the application of inclusion and exclusion criteria, our sample comprised 98 patients.

All the patients had a diagnosis of prostate adenocarcinoma confirmed by transrectal ultrasound-guided biopsy. Gleason scores and prostate-specific antigen (PSA) blood levels were available in all cases. The patients had undergone local exploration by digital rectal examination and transrectal ultrasound, performed by urologists from our hospital with extensive experience in prostate cancer. They had also undergone mpMRI at diagnosis. The findings of the above tests were used to stratify patients into one of the following NCCN risk groups: low risk (T1b–T2a and Gleason score < 7 and PSA < 10 ng/mL), intermediate risk (T2b-T2c or Gleason score 7 or PSA 10–20 ng/mL), or high or very high risk (T3a–T4 or Gleason score 8–10 or PSA ≥ 20 ng/mL). Patients at intermediate risk were considered to have an unfavorable prognosis if they met the following NCCN criteria: two or three intermediate-risk factors and/or Gleason score 7 (4 + 3), and/or ≥50% biopsy core involvement).

Patients diagnosed with intermediate- or high-risk prostate cancer underwent additional staging with computed tomography of the chest, abdomen, and pelvis (CT-CAP) and bone scintigraphy or choline/PSMA positron emission tomography (PET), as determined by the treating physician.

### 2.2. Treatment

All the patients received intensity-modulated RT with doses and volumes varying according to risk. Between 2009 and 2013, patients with intermediate- and high-risk prostate cancer received a dose of 80 Gy delivered in 2-Gy fractions. Following modification of the hospital’s protocol for treating prostate cancer in 2013, the patients received 70.2 Gy in 2.7-Gy fractions (EQD2, 80.03 Gy). The clinical target volume for patients with intermediate and high-risk disease confined to the prostate (stage T1–T2) included the prostate, and in addition, for intermediate risk the CTV included the base of seminal vesicles, and for high-risk the CTV included the entire seminal vesicles. The corresponding volume for patients with locally advanced disease (T3–T4) extended beyond the prostate to cover extracapsular extension [10].

High-risk patients received long-term ADT (24 months), while intermediate-risk patients received short-term ADT (6 months). All patients received injectable luteinizing hormone–releasing hormone agonists, started 2 months before radiotherapy.

### 2.3. Follow-Up

Patients were followed up weekly during RT. On completion of treatment, follow-up visits were held every 3 months for the first 2 years, every 6 months for the next 3 years, and annually thereafter. The visits consisted of clinical history, physical examination, and laboratory tests, including PSA. mpMRI was ordered 6 months after completion of RT to evaluate treatment response; these images were used to calculate post-RT ADC values.

Biochemical recurrence was defined using the Phoenix criteria (PSA nadir + 2 ng/mL) [11]. All patients who experienced biochemical recurrence underwent a prostate mpMRI and CT-CAP + bone scintigraphy or choline/PSMA PET to check for local, locoregional, or distant recurrence [12].

In cases of local and locorregional/distant relapse in the prostate mpMRI and convencional imaging (CT-CAP + bone scintigraphy), a choline/PSMA-PET was needed to confirm both local relapse and locorregional/distant relapse.

In cases of exclusive local relapse by prostate mpMRI and conventional imaging, a prostate biopsy was required.

### 2.4. Multiparametric MRI

The patients underwent a whole-body MRI on a 3.0 T scanner (Signa HDxT-3T; GE Medical Systems, Milwaukee, WI, USA) with an external phased-array coil. They had all fasted and performed bowel preparation to prevent gas-induced artifacts.

The following images were acquired: transverse T1-weighted fast-spin echo (FSE) images (repetition time [TR], 518 ms; echo time (TE), minimum-full; slice thickness, 3 mm; slice gap, 0.3 mm; field of view (FOV), 26 × 26 cm; matrix, 256 × 192 pixels) and fast recovery T2-weighted FSE images in three planes—transverse (FOV, 24 cm; slice thickness, 3 mm; slice gap, 0.2 mm; TE, 180 ms; TR, 4000 ms; matrix, 288 × 288 pixels), coronal (FOV, 27 cm; slice thickness, 4 mm; slice gap, 0.4 mm; TE, 144 ms; TR, 4600 ms; matrix, 332 × 332 pixels), and sagittal (FOV, 29 cm; slice thickness, 4 mm; slice gap, 0.4 mm; TE, 170.24 ms; TR, 4605 ms; matrix, 288 × 288 pixels). The subsequent sequences obtained were a dynamic axial 3D T1 sequence with intravenous contrast (FOV, 30 cm; slice thickness, 4.2 mm; number of slices, 24; TE, in-phase; flip angle, 30; matrix, 256 × 128 pixels) and a post-contrast volumetric 3D T1 sequence with saturation in the axial plane (FOV, 30 cm; slice thickness, 3 mm; number of slices, 84; TE, minimum full; flip angle, 12; matrix, 256 × 256 pixels) and the coronal plane (FOV, 34 cm; slice thickness, 3 mm; number of slices, 68; TE, minimum full; flip angle, 12; matrix, 256 × 256 pixels).

The DW imaging sequence was acquired using a single-shot spin-echo planar imaging sequence with bipolar gradient pulses along three orthogonal axes with the following parameters: TR, 6000–6500 ms; TE, minimum; FOV, 28 cm; slice thickness, 5 mm; acquisition matrix, 128 × 128 pixels, b-values of 0 and 1000 mm^2^/s; no slice gap; acquisition time of approximately 2 min depending on the patient; acquisition encompassing the entire pelvis.

The DW-MRI data were transferred to a commercial workstation and processed using Functool (GE Healthcare, Milwaukee, WI, USA) to calculate the ADC values from the ADC maps. The values were calculated in units of 10^−3^ mm^2^/s by dividing the signal intensity in the regions of interest by 1000.

### 2.5. Image Analysis

Two radiologists with more than 25 and 15 years’ experience with MRI in the diagnosis of genitourinary disorders determined in consensus the location of each tumor using the Prostate Imaging Reporting and Data System. MRI findings for prostate cancer included a focal lesion with high signal intensity on T2-weighted images, which showed low signal intensity on the ADC map and high signal intensity on DW images with a *b*-value of 1000 mm^2^/s, with or without early contrast enhancement and rapid washout on dynamic contrast-enhanced imaging. ADC maps were generated pixel by pixel using the integrated software tool Functool version 2.1. Regions of interest containing as much of the tumor as possible were drawn onto the ADC maps. For tumors visible on multiple slices, ADC values were calculated for each slice, and for patients with multiple lesions, all the lesions were measured. In both cases, the lowest values were taken. For the post-RT ADC calculations, both radiologists in the consensus drew a region of interest at the site of each original tumor. The calculations were made using the same methods as described above.

### 2.6. Statistical Analysis

Statistical analyses were performed in IBM SPSS Statistics version 21.0 (IBM Corp; Armonk, NY, USA).

Biochemical recurrence-free survival, local recurrence-free survival (LRFS), locoregional recurrence-free survival, and distant recurrence-free survival were calculated from diagnosis (date of prostate biopsy) to the corresponding event. Progression-free survival (PFS) was calculated from diagnosis to any of the aforementioned events. Overall survival was calculated from diagnosis to death of any cause, while cancer-specific survival was calculated from diagnosis to death due to prostate cancer.

Differences in ADC values at diagnosis and 6 months after completion of RT were compared overall and by subgroups of patients (absence vs. presence of progression, local recurrence, biochemical recurrence, locoregional recurrence, and distant recurrence).

The patients were divided into quartiles based on relative changes in ADC values from diagnosis to 6 months post-RT. Survival was compared between patients with values above Q3 and those with values between Q1 and Q3.

Receiver operating characteristic (ROC) analysis was performed to identify optimal ADC cutoff values for predicting 10-year PFS and 10-year LRFS. With these cutoffs, Kaplan–Meier curves were created to compare PFS and LRFS between the two groups of patients.

Potential relations were studied using the *t* test or Mann–Whitney U test for quantitative variables and the chi-square or Fisher exact test for qualitative variables.

Multivariate analyses were performed using Cox proportional hazards regression models and included potential factors: age at diagnosis, initial ADC, ADC value post-RT, T-stage, Gleason score, risk groups.

## 3. Results

In total, 124 patients diagnosed with prostate cancer at our hospital were treated with RT and ADT and underwent post-RT mpMRI between 2008 and 2019. Out of the 110 patients who underwent mpMRI scans 6 months after completing RT, 98 had sufficient data to calculate tumor ADC values.

Ninety-eight patients met all the inclusion criteria and none of the exclusion criteria were included in the study. Their characteristics are summarized in Table 1.

ADT was administered for 6 months in 26 patients (26.5%) and 24 months in 69 (70.4%). It was discontinued due to poor tolerance in three patients (3.1%) (after 9, 12, and 18 months). Forty-seven patients (47.9%) received 80 Gy, fourty-eight (48.9%) 70.2 Gy, two (2.0%) 76 Gy, and one (1.0%) 78 Gy.

After a mean ± SD follow-up of 95.36 ± 30.54 months, 10-year survival rates were 76.5% for biochemical recurrence-free survival, 75.6% for PFS, 93.8% for LRFS, 85.5% for distant recurrence-free survival, and 89.5% for overall survival.

Nineteen patients (19.4%) experienced progression, seventeen (17.7%) biochemical recurrence, four (4.1%) local recurrence, ten (10.2%) locoregional recurrence, thirteen (13.3%) local or locoregional recurrence, and nine (9.2%) distant recurrence. Ten patients (10.2%) died, and one (1.0%) due to prostate cancer.

All four patients with local recurrence were diagnosed by mpMRI + biopsy or mpMRI+ PSMA-PET in case of local + locorregional/metastasis relapsed.

The mean ADC value was 0.81 ± 0.18 × 10^−3^ mm^2^/s at diagnosis and 1.30 ± 0.18 × 10^−3^ mm^2^/s 6 months after RT (*p* < 0.001). This change represented a mean increase of 70.1% ± 46.8%. Changes in ADC values ranged from −10.6% to 34.5% in 25% of patients and from 34.5% to 97.5% in the middle 50%. The remaining 25% had an increase of more than 97.5%.

**Figure 1:**
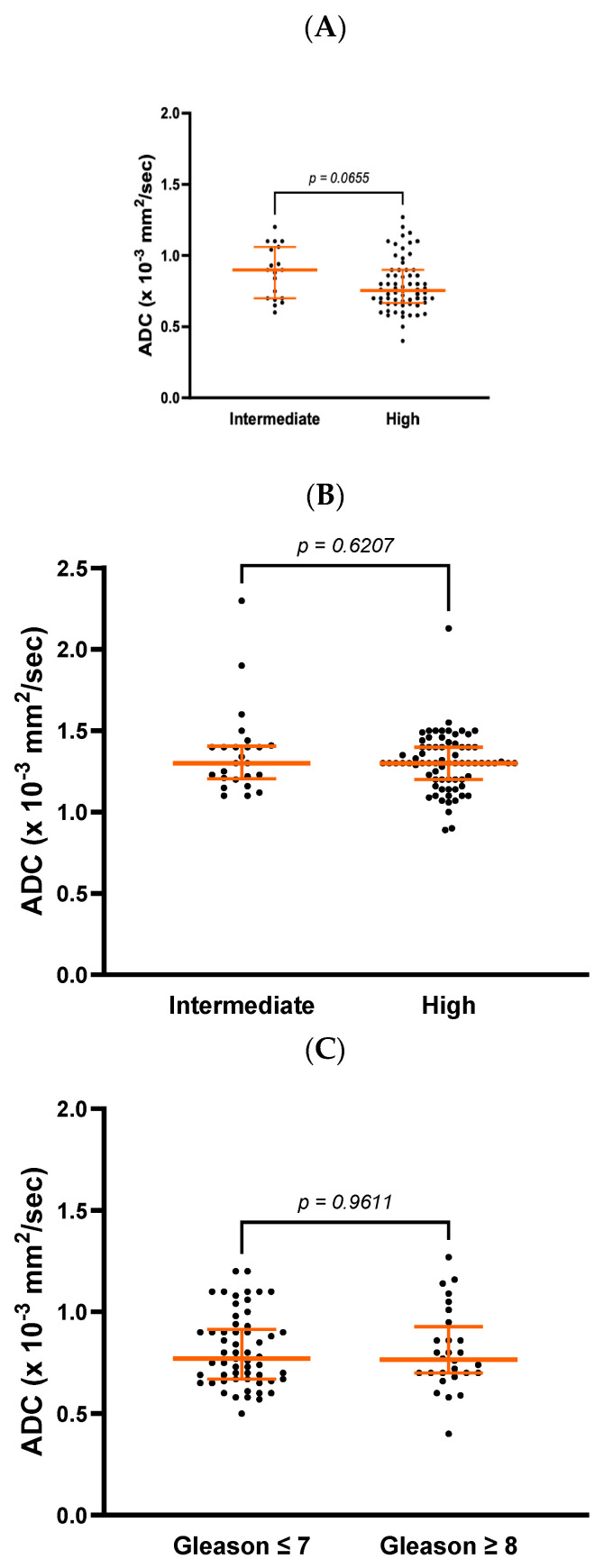

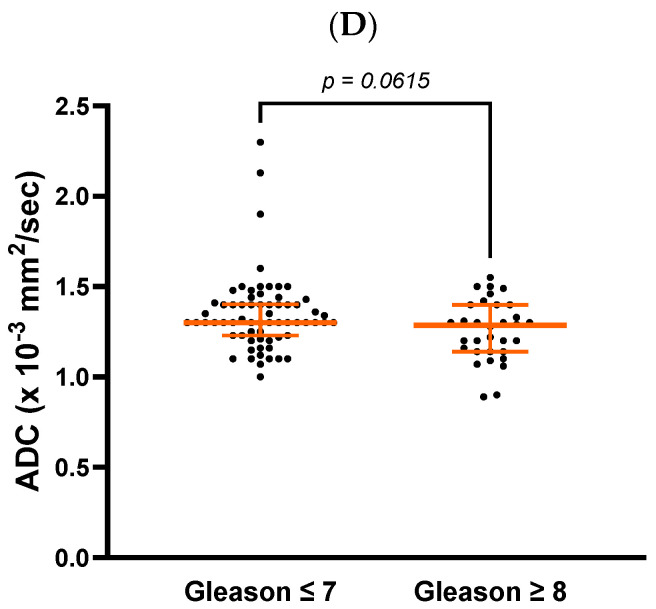
Differences in ADC values at diagnosis and post-RT between patients with intermediate- and high-risk prostate cancer (**A**,**B**) and patients with a Gleason score ≤ 7 and ≥8 (**C**,**D**).

There was a trend towards a difference in ADC values at diagnosis between patients with intermediate-risk and high-risk prostate cancer (0.9 ± 0.4 vs. 0.8 ± 0.2 × 10^−3^ mm^2^/s, *p* = 0.065) but no differences in post-RT values (1.3 ± 0.2 vs. 1.3 ± 0.2 × 10^−3^ mm^2^/s, *p* = 0.627, Figure 1A,B). On comparing patients with a Gleason score ≤ 7 vs. ≥8, there were no differences in ADC values at diagnosis (0.77 ± 0.25 vs. 0.77 ± 0.23 × 10^−3^ mm^2^/s, *p* = 0.961, Figure 1C). A trend, however, was observed 6 months after completion of RT (1.30 ± 0.17 vs. 1.29 ± 0.26 × 10^−3^ mm^2^/s, *p* = 0.0615, Figure 1D).

mpMRI was performed a median of 6.00 [5.00–6.00] months after completion of RT. No significant differences were observed in the timing of this test between patients who experienced progression and those who did not (*p* = 0.410).

No significant differences in ADC values at diagnosis were observed between patients with and without progression (0.77 ± 0.28 vs. 0.74 ± 0.21 × 10^−3^ mm^2^/s, *p* = 0.974) or between those with and without local recurrence (0.77 ± 0.25 vs. 0.65 ± 0.15 × 10^−3^ mm^2^/s, *p* = 0.211). There were also no differences in ADC values at diagnosis according to the presence or absence of biochemical recurrence, locoregional recurrence, or distant recurrence.

Mean post-RT ADC values were significantly lower in patients who experienced local recurrence (1.09 ± 0.18 vs. 1.30 ± 0.20 × 10^−3^ mm^2^/s, *p* = 0.020) and those who experienced any form of progression (1.23 ± 0.20 vs. 1.30 ± 0.21 × 10^−3^ mm^2^/s, *p* = 0.04).

Patients with biochemical recurrence also had a significantly lower post-RT ADC value than those who did not (1.20 ± 0.14 vs. 1.34 ± 0.21 × 10^−3^ mm^2^/s, *p* = 0.004). Locoregional recurrence was associated with a trend towards a lower post-RT ADC value (1.22 ± 0.14 vs. 1.33 ± 0.21 × 10^−3^ mm^2^/s, *p* = 0.088). This value was also lower in patients with local or locoregional recurrence (1.19 ± 0.14 vs. 1.34 ± 0.20 × 10^−3^ mm^2^/s, *p* = 0.004). No differences were observed between patients with and without distant recurrence (1.22 ± 0.16 vs. 1.33 ± 0.20 × 10^−3^ mm^2^/s, *p* = 0.138).

**Figure 2:**
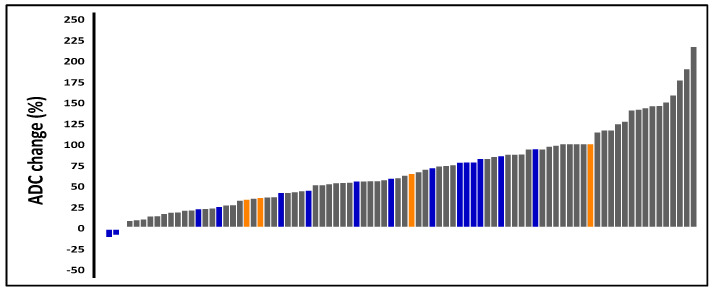
Relative changes in ADC values from diagnosis to post-RT. The figures show patients with local recurrence (orange) and any form of progression (blue).

Patients whose ADC values increased by more than 97.5% from diagnosis to post-RT had a lower risk of progression than those with a smaller increase (4.76% vs. 26.56%, *p* < 0.001) (Figure 2).

**Figure 3:**
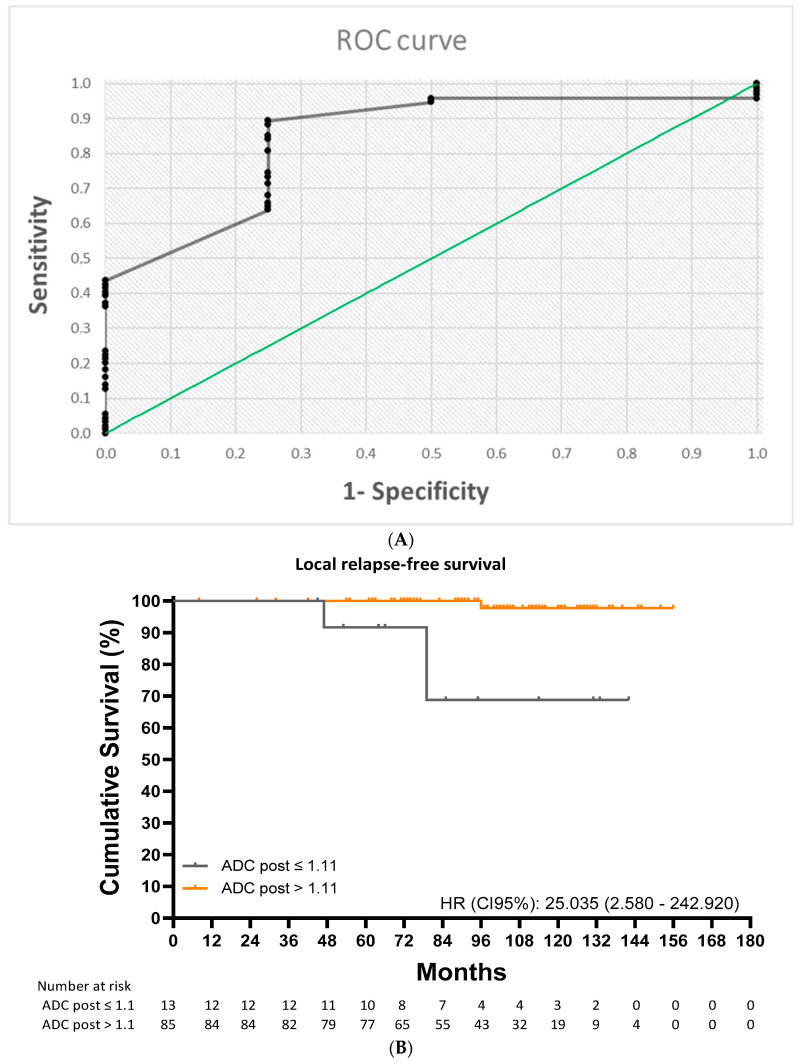
(**A**) Receiver operating characteristic analysis for local recurrence-free survival. (**B**) Kaplan–Meier curves for progression-free survival in patients with an ADC value ≤ 1.11 vs. >1.11 × 10^−3^ mm^2^/s.

ROC analysis identified an ADC cutoff value of 1.11 × 10^−3^ mm^2^/s for predicting local recurrence (area under the curve [AUC] = 0.843; 95% CI: 0.660–1.00; *p* < 0.001) with a sensitivity of 89.4%, a specificity of 75.0%, a positive predictive value (PPV) of 98.8%, and a negative predictive value (NPV) of 23.1% (Figure 3A). Patients with an ADC value at or below the cutoff of 1.11 × 10^−3^ mm^2^/s) had a 10-year LRFS of 66.8% compared with 97.7% for those with a higher value (HR: 25.04 [2.58–242.92]; *p* < 0.001) (Figure 3B).

**Figure 4:**
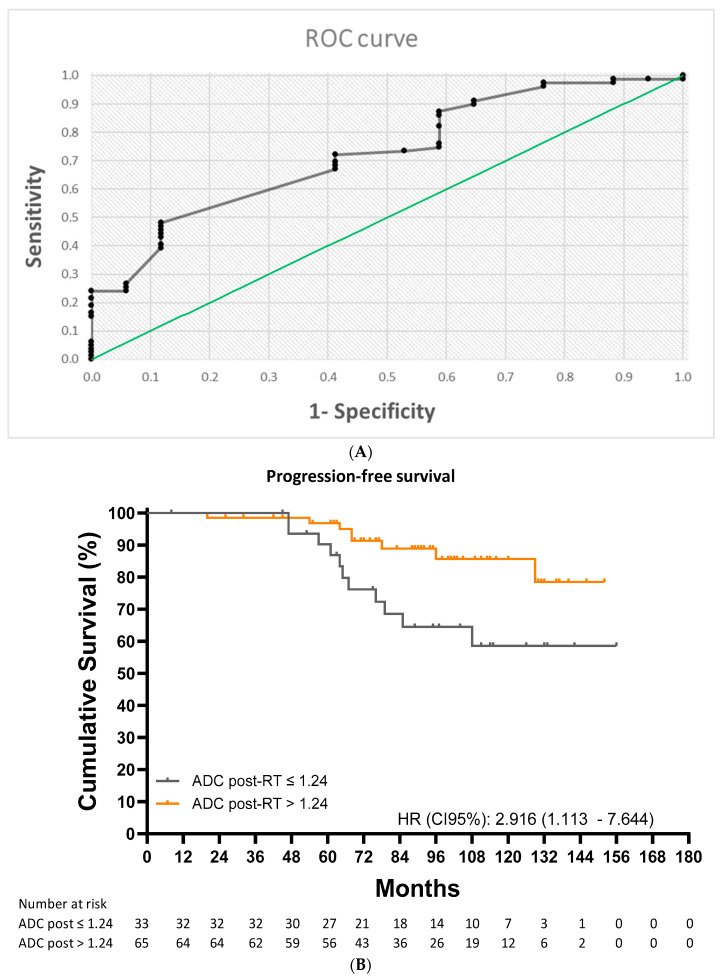
(**A**) Receiver operating characteristic analysis for progression-free survival. (**B**) Kaplan–Meier curves for progression-free survival in patients with an ADC value ≤ 1.24 vs. >1.24 × 10^−3^ mm^2^/s.

The ROC analysis identified a post-RT ADC cutoff value of 1.24 × 10^−3^ mm^2^/s (AUC = 0.713; 95% CI: 0.593–0.833; *p* < 0.001) for predicting any form of disease progression (Figure 4A). This cutoff had a sensitivity of 72.2%, a specificity of 57.9%, a PPV of 87.7%, and an NPV of 33.3%. Ten-year progression-free survival (PFS) was 58.6% for patients with a value at or below the cutoff and 85.6% for those with a value above the cutoff (HR: 2.92 [1.11–7.64]; *p* = 0.015) (Figure 4B).

In the univariate analysis, a post-treatment ADC value ≤ 1.11 × 10^−3^ mm^2^/s was a significant prognostic factor for a lower LRFS (HR: 25.103 [2577–250] p = 0.006). In the multivariate analysis, cox proportional hazards regression models confirm that after adjustment for covariates, a post-treatment ADC value ≤ 1.24 × 10−3 mm2/s was a significant prognostic factors for a lower PFS (HR: 3823 [1371–10,657], p = 0.010).

## 4. Discussion

To our knowledge, this is the first study to show that post-RT ADC values derived from mpMRI can predict local recurrence in patients with prostate cancer treated with RT and ADT. Moreover, this long follow-up study shows the value of ADC as a predictive factor of disease progression in patients treated with RT and ADT.

In our study, all the patients had a diagnosis of prostate adenocarcinoma confirmed by transrectal ultrasound-guided biopsy [13] with a Gleason score and PSA levels in all cases. Also, all the patients had undergone local exploration by digital rectal examination and transrectal ultrasound, and all patients had also undergone mpMRI at diagnosis before starting the ADT and radiotherapy treatment.

Several studies have reported changes in ADC values following treatment with ADT [14] or RT [15,16,17], and there have even been reports of increases just days after completion of RT [18], probably based on post-radiation changes, making a relatively low cellularity associated with variable degrees of glandular atrophy, fibrosis, and inflammatory reaction that are characterized by an increase in diffusion distance of water protons with a subsequent increase in their ADC values.

These changes in ADC are shown in different studies such as Liu et al. [9], a study published in 2014 of 78 patients with high-risk prostate cancer treated with RT and ADT, reported an increase in mean ADC from 1.04 × 10^−3^ mm^2^/s at diagnosis to 1.45 × 10^−3^ mm^2^/s post-RT. In 2023, Onal et al. [6] reported a mean increase of 40.5% from diagnosis (0.76 × 10^−3^ mm^2^/s) to post-treatment (1.09 × 10^−3^ mm^2^/s). The study involved 229 patients with low- and intermediate-risk prostate cancer treated exclusively with RT. In our study, mean ADC increased from 0.81 × 10^−3^ mm^2^/s at diagnosis to 1.30 × 10^−3^ mm^2^/s post-RT (mean increase of 70.11%).

Although the above studies showed quantitative changes in ADC values after treatment with RT and ADT, the significance of these changes and their potential use as predictors of treatment response require further investigation.

We observed a significant difference in post-RT ADC values between patients who experienced local recurrence and those who did not (1.09 ± 0.18 vs. 1.30 ± 0.20 × 10⁻^3^ mm^2^/s, *p* = 0.020), supporting previous findings by Liu et al. [9] (1.27 ± 0.14 × 10⁻^3^ vs. 1.49 ± 0.12 × 10⁻^3^ mm^2^/s, *p* = 0.001).

We also observed a difference in post-RT ADC values between patients with and without any form of progression (1.23 ± 0.20 vs. 1.30 ± 0.21 × 10⁻^3^ mm^2^/s, *p* = 0.004). In this case, our findings are similar to those reported by Onal et al. [6], who reported a value of 0.94 ± 0.07 × 10⁻^3^ mm^2^/s for patients with recurrence and 1.10 ± 0.20 × 10⁻^3^ mm^2^/s for those without (*p* < 0.001).

Our findings, together with those of Liu et al. [9] and Onal et al. [6], show that patients with prostate cancer who experience local recurrence or disease progression have lower post-RT ADC values than those whose disease does not recur. Having a cutoff for identifying patients at risk of recurrence would be very valuable.

Our study is the first to explore the value of post-RT ADC as a predictive factor of local recurrence. The cutoff identified, 1.11 × 10⁻^3^ mm^2^/s, predicted local recurrence with a sensitivity of 89.92% and a PPV of 98.82%. Patients with a value above this cutoff were much more likely to be free of local recurrence at 10 years than those with a value at or below the cutoff (97.7% vs. 66.8%; HR: 25.04 [2.58–242.92]; *p* < 0.001). The association between post-RT ADC and local recurrence is one of the most novel findings in our study, as it shows, for the first time, that ADC values derived from mpMRI can be used to identify patients at an increased risk of local recurrence. The need for further investigation in this area was highlighted by Onal et al. [6] in 2022. Our observation of a significant association between post-RT ADC values and local recurrence has significant clinical and prognostic implications. It is particularly relevant considering that patients with high-risk prostate cancer have an increased risk of local recurrence [19] and that significant associations have been observed between local recurrence and overall, cancer-specific, and metastasis-free survival following RT in this setting [20,21].

We also identified a post-RT ADC cutoff value that predicted overall recurrence with a sensitivity of 72.2% and a PPV of 87.72%. In this case, 10-year PFS was 58.6% for those with a value at or below the cutoff (1.24 × 10⁻^3^ mm^2^/s) and 85.6% for those with a value above the cutoff. Liu et al. [17] reported a similar cutoff for recurrence (1.34 × 10⁻^3^ mm^2^/s), although their median follow-up time was shorter than ours (40.00 vs. 95.36 months). Onal et al. [6] identified a cutoff of 0.96 × 10⁻^3^ mm^2^/s for predicting biochemical recurrence, but this was in patients with low- and intermediate-risk prostate cancer followed for 80.00 months.

Patients with an increase of more than 97.5% in post-RT ADC values from diagnosis had a lower risk of progression than those who experienced a smaller increase (4.76% vs. 26.56%, *p* < 0.001). This finding indicates that patients with substantial increases in ADC values after RT respond well to treatment and have a lower risk of recurrence.

ADC values could vary for numerous reasons, including differences in risk groups, Gleason score, timing of mpMRI, and use of ADT. These reasons could explain the differences in ADC values between our study and the results from Liu et al. [9] and Onal et al. [6].

Differences in post-RT ADC values: In our study these values are lower than those reported by Liu et al. [9]—who studied patients exclusively with high-risk prostate cancer—and higher than those reported by Onal et al. [6]—who studied patients with low- and intermediate-risk prostate cancer. This can be explained by the differences in ADC values according to risk profile, which may be linked to correlations observed between Gleason scores and relative increases in ADC values following treatment with RT. Iraha et al. [22], for example, found that patients with higher Gleason scores experienced greater increases in ADC values after treatment. Although in our study we found no clear differences in ADC values between patients with Gleason ≤ 7 and ≥8.

Differences in cut-off values: In our study, mpMRI was performed on average 6 months after completion of RT, compared with 2 months in the study by Liu et al. [17] and 4 months in that of Onal et al. [6]. Treatment is another factor that can influence ADC measurements. Patients were treated with neoadjuvant (2–3 months), concomitant, and adjuvant ADT with RT in our series, concomitant and adjuvant ADT with RT in the study by Liu et al. [17], and RT only in the study by Onal et al. [6].

The findings of this study suggest that ADC values can be used as an early indicator of response to combination therapy with RT and ADT in patients with intermediate- and high-risk prostate cancer. This would have important implications for both follow-up and treatment. On the one hand, it would help select patients who might benefit from closer clinical monitoring, such as more frequent PSA testing and periodic mpMRI scans to rule out local recurrence. On the other, it could be used to select patients who could potentially benefit from treatment intensification. Several treatment-intensification strategies with antiandrogens are being studied in numerous clinical trials, such as ATLAS [23] and THUNDER [24]. The FLAME [25] and ASCENDE [26] trials, in turn, have shown the benefits of dose-escalated RT and prolonged ADT.

Our study has some limitations, including those inherent to any study with a retrospective design. ADC calculations are operator dependent and prone to error. To address this potential source of bias, ADC values were calculated by two radiologists with extensive experience in prostate cancer. It is also important to note that ADC values change with time. One strength of this study is that the mpMRI scans used to calculate these values were performed at a similar point following completion of RT in all patients (6.00 [5.00–6.00] months]. Another strength is the long follow-up time. By following patients for a mean of 95.36 months, we were able to detect most recurrences.

## 5. Conclusions

This is the first study to show that post-RT ADC values are predictive of local recurrence in patients with intermediate- and high-risk prostate cancer treated with RT and ADT. Moreover, this long-term study shows that post-RT ADC values could be used as an early predictive factor of treatment response in patients with prostate cancer treated with RT and ADT. These findings should be confirmed in a prospective setting and may help to select patients at higher risk of local relapse or any progression who could benefit from a closer follow-up and treatment escalation.

## Figures and Tables

**Table 1:** Characteristics of patients included in the study.

		*n* = 98
**Age, mean**		72 [67–76]
**Diagnosis PSA ng/mL**		10.15 [6.93–21]
**Gleason**		
	Gleason 6, *n* (%)	14 (14.29)
	Gleason 7, *n* (%)	52 (53.06)
	Gleason 8, *n* (%)	25 (25.51)
	Gleason 9, *n* (%)	7 (7.14)
**T Stage**		
	T1–T2a, *n* (%)	13 (13.27)
	T2b–T2c, *n* (%)	38 (38.78)
	T3–T4, *n* (%)	47 (47.95)
**Risk group**		
	Intermediate, *n* (%)	25 (25.51)
	High or very high, *n* (%)	73 (74.49)
**Radiotherapy dose (fractions)**		
	70.2 Gy (26), *n* (%)	48 (48.98)
	76 Gy (38), *n* (%)	2 (2.04)
	78 Gy (39), *n* (%)	1 (1.02)
	80 Gy (40), *n* (%)	47 (47.69)
**Androgen deprivation therapy**		
	6 months, *n* (%)	26 (26.53)
	9 months, *n* (%)	1 (1.02)
	12 months, *n* (%)	1 (1.02)
	18 months, *n* (%)	1 (1.02)
	24 months, *n* (%)	69 (70.41)

## Data Availability

All data generated or analyzed during this study are included in this published article.

## Abbreviations

ADT: Androgen Deprivation Therapy; AUC: Area Under Curve; HR: Hazard Ratio; LRFS: Local Recurrence-Free Survival; mpMRI: Multiparametric Magnetic Resonance Imaging; PCa: Prostate Cancer; PFS: Progression-Free Survival; PPV: Positive Predictive Value; ROC: Receiver Operating Characteristic; RT: Radiotherapy; SD: Standard Deviation; DRE: Digital rectal examination ADC: Apparent diffusion coefficient; DW: Diffusion-weighted; NCCN: National Comprehensive Cancer Network; PSA: Prostate-specific antigen; CT-CAP: Computed tomography of the chest, abdomen, and pelvis; PET: Positron emission tomography; PSMA: Prostate-specific membrane antigen; Gy: Gray; EQD2: Equivalent dose in 2 Gy fractions; CTV: Clinical target volume; TR: Repetition time; TE: Echo time; FOV: Field of view; DW-MRI: Diffusion-weighted magnetic resonance imaging; NPV: negative predictive value.
